# Validation of the Arabic version of the Launay-Slade Hallucination Scale Extended: A population-based online survey in Saudi-Arabia

**DOI:** 10.1371/journal.pone.0341864

**Published:** 2026-02-11

**Authors:** Haya Althuwaini, Georg Meyer, Ryan Ward, Henry Meyer

**Affiliations:** 1 Department of Radiology, Faculty of Health and Rehabilitation Sciences, Princess Nourah bint Abdulrahman University, Riyadh, Saudi Arabia; 2 Department of Psychology, Faculty of Health and Life Sciences, University of Liverpool, Liverpool, United Kingdom; 3 IDEAS, Digital Innovation Facility, University of Liverpool, Liverpool, United Kingdom; 4 Hanse Wissenschaftskolleg, Delmenhorst, Germany; 5 Department of Computer Science and Mathematics, Liverpool John Moores University, Liverpool, United Kingdom; 6 Faculty of Governance and Global Affairs, Leiden University, The Hague, The Netherlands; Northumbria University, UNITED KINGDOM OF GREAT BRITAIN AND NORTHERN IRELAND

## Abstract

Hallucinations occur when individuals perceive sensory events as real despite the absence of external stimuli. The Launay-Slade Hallucination Scale Extended Version (LSHS-E) is a validated measure for assessing hallucination proneness and has been adapted into several languages. Numerous studies worldwide have examined the factor structure of the LSHS-E, yet none have focused on the Arabic language. This study, conducted in Saudi Arabia, aimed to validate the Arabic version of the LSHS-E and explore its factor structure in an Arabic-speaking population. Following translation and back-translation, the Arabic LSHS-E was distributed to a Saudi Arabian general population sample (n = 428) via the Qualtrics Platform. Reliability was confirmed with high internal consistency (Cronbach’s alpha = 0.916; 95% CI: 0.904–0.927). Confirmatory factor analysis indicated that a four-factor model—comprising intrusive thoughts, vivid daydreams, multisensory HLEs, and auditory and visual HLEs—provided the best fit for the data (CFI = 0.93, RMSEA = 0.07, SRMR = 0.05). Positive correlations between LSHS-E scores and psychotic symptoms measured by the positive subscale of the Community Assessment of Psychic Experiences (PCAPE) supported convergent validity (r_s_ = 0.55, p < 0.001). Sociodemographic analyses revealed that younger age (β = −5.30, p = 0.028) and lower income (β = −6.92, p = 0.028)were significant predictors of higher hallucination proneness scores. Our findings reveal response patterns and factor structures consistent with those observed in other languages and cultural contexts. The validated Arabic LSHS-E provides a reliable tool for studying hallucination proneness in Arabic-speaking populations.

## Introduction

Hallucinations can be defined as perception-like experiences that occur without any physical external stimulus, which carry the same intensity as real perceptions, and which cannot be consciously controlled by the person experiencing them [1]. They may occur in any sensory domain including auditory, visual, olfactory, gustatory, or tactile modalities [2]. Hallucinations are commonly associated with mental illness, particularly schizophrenia [3–5]. Experiencing hallucinations, however, is not uncommon among healthy individuals, suggesting that such experiences can occur in the general population without necessarily indicating a mental health condition [6–10]. The prevalence of hallucinatory experiences in the non-clinical population is generally estimated at around 12.7% [11], though lower estimates, such as 3% in older adults [12], and higher rates up to 38.7% [13] have also been reported.

Research on hallucinatory-like experiences (HLEs) in non-clinical population is essential for understanding the distinction between normal perceptual phenomena and those that suggest a higher risk of developing mental disorders [2]. It would also allow for the early identification of hallucinations and the assessment of their correlates without the confounding factors often found in clinical samples, such as medication, self-selection, comorbidities, and stigma [14]. The Launay-Slade Hallucinations Scale (LSHS), originally introduced as a 12-item questionnaire by Launay and Slade (1981), is one of the most widely used instruments for assessing HLEs. It captures both pathological and sub-clinical hallucinations, based on the idea that hallucinatory experiences exist on a continuum that ranges from normal mental states to mental illness [15]. Over the years, the LSHS has undergone several revisions in terms of content, the number of items included and the response format, shifting from simple yes/no answers to a Likert scale [7,9,16,17].

The scale demonstrates a multi-dimensional approach in the tendency to hallucinations and is widely used in research across various populations, including both healthy individuals [16,18–20] and clinical groups, such as those with Alzheimer’s disease [21] and schizophrenia [20,22,23]. It serves multiple purposes, for example, exploring the relationship between childhood memories and hallucination proneness [24], and investigating the neural basis of hallucinations through neuroimaging studies [3,25,26].

The LSHS was initially developed in English [15] and has since been adapted across various languages and populations, consistently demonstrating its reliability and validity [19,27–29]. However, different response formats and item numbers have led to various factorial structures, including two-factor [17,22,30], three-factor [18,27,31], four-factor [14,16,28,29,32,33], and five-factor [9] models.

In our study, LSHS-Extended (LSHS-E) version was used, which has been validated in English as well as in multiple languages through cross-national studies [29]. Similar response patterns and factor analyses have been observed in diverse cultural settings, such as India [28], Italy [33], and Spain [32]. However, no validation study for an Arabic version has been identified, which is the gap we aim to address. This research, conducted in Saudi Arabia, tested the psychometric properties of the Arabic LSHS-E. We sought to explore the dimensionality of HLEs using confirmatory factor analysis (CFA) and hypothesised that the four-factor model observed in previous studies would be replicated in Arabic-speaking populations.

## Methodology

### Procedure

This cross-sectional study utilized an online survey for data collection through the Qualtrics platform (available at https://www.qualtrics.com/). The survey was distributed to a Saudi Arabian population, and participants were required to provide electronic informed consent form by clicking an agreement box on the first page before proceeding. An information sheet was also provided, explaining the purpose of the study, which was to assess hallucination experiences among healthy individuals in Saudi Arabia. Ethical approval for the study was granted by Princesses Nourah bint Abdulrahman University in Riyadh, Saudi Arabia [IRB log number 21-0312], and by the central research ethics committee at the University of Liverpool in the UK [ref number 10211].

### Participants

The survey link, which included an Arabic version of the LSHS-E and the positive subscale of the Community Assessment of Psychic Experiences (PCAPE), was distributed online and responses were collected between 1^st^ October 2021 and 15^th^ December 2021. Recruitment was conducted primarily through Saudi X (formerly Twitter) accounts, and the study advertisement explicitly stated that only Saudi individuals aged 18 years or older were eligible to participate. While it was not possible to independently verify nationality or residence for each respondent, the recruitment strategy was targeted at the Saudi population. Online recruitment via social media targeting a specific population has also been successfully employed in previous studies investigating hallucination proneness, such as the Spanish study that used the Spanish version of the LSHS-E [32].

In total, 655 responses were received, of which 144 were incomplete and excluded, leaving 511 complete responses. Individuals who reported drug or alcohol use (n = 14), psychological disorders (n = 62), or neurological disorders (n = 17) were excluded, as the focus of the study was on healthy individuals from the general population. This approach was applied to minimise potential confounding, since hallucinations in these groups may be related to underlying psychopathology or substance use rather than reflecting hallucination proneness in non-clinical populations. These exclusion criteria are consistent with previous research on hallucination proneness in non-clinical samples [28,32]. Some participants answered ‘Yes’ to more than one exclusion criterion (e.g., both psychological and neurological disorders). After applying these exclusion criteria, the final analytic sample comprised 428 participants. Participation was voluntary, with no financial or other compensation provided.

### Measures

#### Launay-Slade Hallucinations Scale-Extended (LSHS-E) [9].

The LSHS-E is a self-report scale widely used to assess predisposition to hallucinations in the general population [16]. It consists of 16 questions that explore hallucinatory experiences across visual, auditory, olfactory, and tactile modalities. Additionally, the scale includes items related to hypnagogic and hypnopompic hallucinations, vivid daydreams, and the experience of sensed presence (S1 Appendix). Responses are rated on a Likert scale ranging from zero ‘certainly does not apply to me’ to four ‘certainly applies to me’. A higher overall score indicates a greater likelihood of being prone to hallucinations [28,29].

#### Community Assessment of Psychic Experiences (CAPE) [34].

The CAPE, which has been utilized in various languages (available at https://cape42.homestead.com/), is designed to measure psychosis proneness in both clinical and research contexts. This 42-item self-report tool assesses three dimensions: Positive (20 items), Negative (14 items), and Depressive (8 items) psychotic experiences [34,35]. For this study, we used a brief version of the CAPE, specifically the 20-item PCAPE (S2 Appendix) [36]. Each question was answered on a 4-point Likert scale ranging from ‘never’ to ‘nearly always’.

#### Translation process.

Both scales were initially translated into Arabic by the researcher with the assistance of a certified clinical psychologist. Subsequently, the scales were blindly translated by two other researchers. The translation process was discussed through online group meetings until the final Arabic version was approved. To verify accuracy, the scales were back translated into English by an independent researcher [28]. A pilot study tested the Arabic-adapted version before distribution. All team members were native Arabic speakers and fluent in English, holding tertiary educational qualifications obtained through training in English-speaking countries. The Arabic version of both scales is included in the Supporting Information - S1 and S2 Appendices.

### Statistics

Statistical analyses were conducted using Statistical Package for the Social Sciences (IBM SPSS) version 27 [37] and the R programming language [38]. All tests were two-tailed with a significance threshold set at p < 0.05, adjusted for multiple comparisons where appropriate. The reliability of the scales was assessed using Cronbach’s alpha.

To examine the factor structure, CFA was carried out using the ‘lavaan’ [39] and ‘semTools’ [40] packages in R. This analysis aimed to determine whether the LSHS-E data conformed to the commonly reported four-factor structure of the LSHS. We also analysed the factor structure using exploratory factor analysis (EFA). The statistical test and results are provided in the Supporting Information (S4 Appendix, and S1–S3 Tables for interested readers).

To evaluate convergent validity, correlational analysis between the LSHS-E, its four factors (intrusive thoughts, vivid daydreams, multisensory HLEs and auditory-visual HLEs) and PCAPE were performed.

To investigate the effect of sociodemographic factors on hallucination-proneness, linear regression models were used with the total LSHS-E score as the dependent variable. Predictor variables included gender, age group, financial status, average income, social status, education level, and professional status. Each demographic variable was entered into separate univariate models to assess its independent relationship with LSHS-E scores. In line with previous studies (e.g., Sahu et al., 2020), sociodemographic variables were treated as categorical and recoded into broader categories to avoid small cell sizes, increase statistical power [41] and improve the stability and interpretability of the regression models (see Table 1; also, the original and transformed sociodemographic coding are provided in the Supporting Information - S3 Appendix). These transformations (e.g., collapsing age into three groups and education into three levels) were implemented using an R script to prepare the data for analysis. Participants without independent financial income were not asked about their income, and this variable was coded as ‘unknown’ in the dataset.

**Table 1:** Demographic data (n = 428), and their relationship with hallucination proneness (LSHS-E).

Variables	Category	Frequency (N)	Percent (%)	Linear regression			
				B	F	*p*_*uncorrected*_	*p*_*corrected*_
Gender	Female	314	73	0.08	0.00	0.957	1.00
	Male	114	27				
Age (years)	18–35	173	41	−5.30	5.68	0.004**	0.028*
	36–55	211	49				
	56 or above	44	10				
Social status	Married	334	78	3.24	3.93	0.048*	0.336
	Single, separated or widowed	94	22				
Employment status	Employed or free business	215	50	1.95	2.06	0.152	1.00
	Unemployed, student or retired	213	50				
Education status	Up to middle school	16	4	−3.85	0.65	0.522	1.00
	High school	80	19				
	Bachelor or higher	332	77				
Independent financial status	Yes	279	65	3.50	6.09	0.014*	0.098
	No	149	35				
Personal monthly income (n = 279, 65%)	9000 SR or below	97	35	−6.92	5.64	0.004**	0.028*
	9001–19000 SR	123	44				
	Above 19000 SR	59	21				

p values were corrected for multiple comparisons using the Bonferroni method (p < .05). B, regression coefficient corresponds to the main contrast for binary predictors, or to the largest effect contrast for predictors with three categories. Reference categories were: female, 18–35 years, up to middle school, married, employed/free business, ≤ 9000 SR, and yes for financial independence.

## Results

### Sociodemographic data

All 428 participants completed the questionnaire, including the scale items and sociodemographic questions, except for ‘average personal monthly income’. This variable was collected only from participants who reported having an independent financial income (n = 279). Participants who responded ‘No’ to this screening question were not asked to provide income data. The sample was predominantly female. Nearly half of the participants were in the middle-age group. The majority were married and held a bachelor’s degree or higher (see Table 1 for details).

### LSHS-E scores

**Fig 1:**
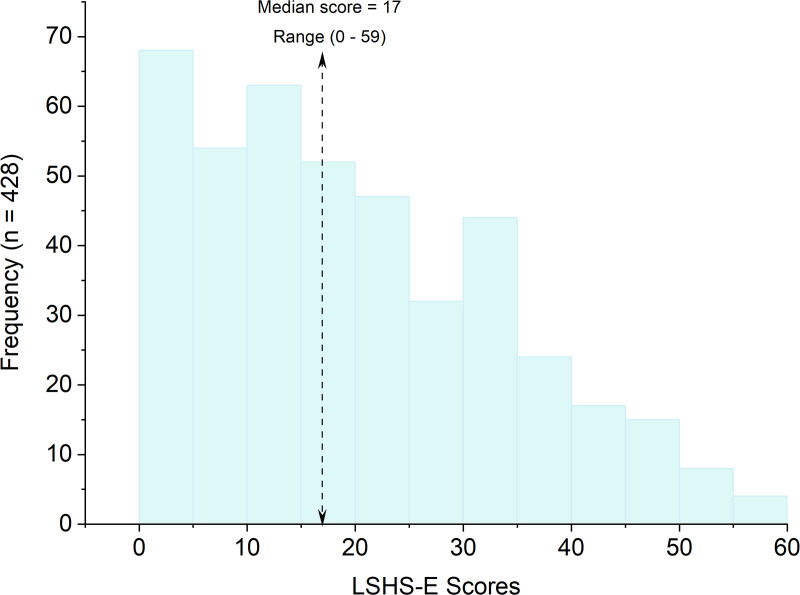
LSHS-E scores distribution (n = 428).

The distribution of LSHS-E scores exhibited positive skewness, as evident from visual inspection of the histogram (Fig 1). This significant deviation from normality was confirmed by a Shapiro-Wilk test (*W* (428) =0.95, *p* < 0.001). Scores on the LSHS-E ranged from 0 to 59, with the median score being 17.

**Fig 2:**
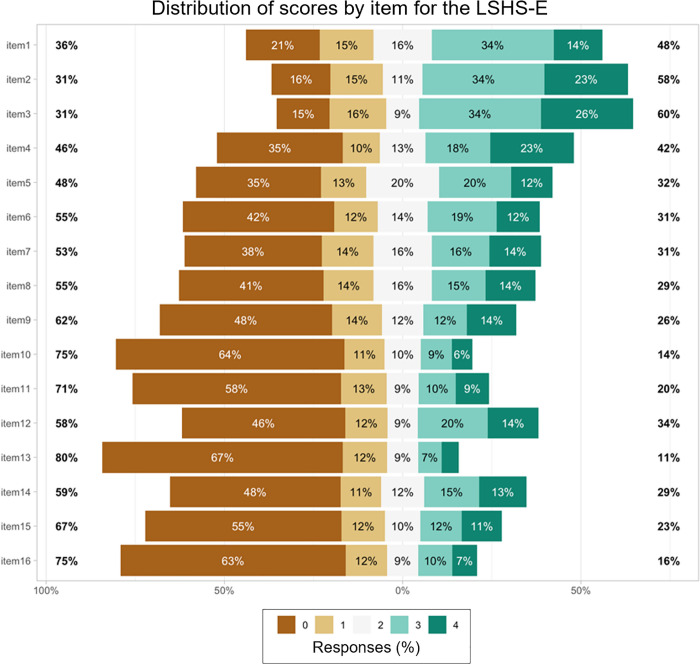
Distribution of LSHS-E scores by item (n = 428). Answers range from 0-4 [certainly does not apply to me “مؤكد انها لاتنطبق علي” – certainly applies to me “مؤكد انها تنطبق علي”].

The proportion of participants selecting ‘possibly applies to me’ [3] or ‘certainly applies to me’ [4] responses on the LSHS-E varied from 11% to 60% depending on the assessed experience (Fig 2). Notably, the most reported hallucinatory experience in this sample is ‘intrusive thoughts’ (items 1, 2 and 3), consistent with findings from Sahu et al. (2020) and Vellante et al. (2012). In contrast, visual hallucinations (items 10 and 16) were reported the least frequently. S1 Appendix in the Supporting Information contains the list of items in both Arabic and English format.

### Reliability

Both the Arabic versions of LSHS-E and PCAPE exhibited excellent internal consistency. The Cronbach’s alpha for LSHS-E was α = 0.92 (95% CI: 0.90 to 0.93), while for PCAPE, it was α = 0.84 (95% CI: 0.82 to 0.86). It’s worth noting that recommended values for internal consistency are typically 0.7 or higher, indicating the robustness of both scales [42,43].

### Confirmatory factor analysis

Due to a violation of the multivariate normality assumption as indicated by Mardia’s test (skew = 43.95, p < 0.001, kurtosis = 374.82, p < 0.001), maximum likelihood estimation with robust standard errors and the Satorra-Bentler scaled test statistic was employed for the CFA models. This method is robust against violations of normality [44]. The models were compared using standard fit indices [45] as well as information criteria, including the Akaike Information Criterion (AIC) [46] and the Bayesian Information Criterion (BIC) [47]. McDonald’s omega was additionally computed from the CFA models. This coefficient incorporates both the magnitude of item-factor associations and the influence of item-specific error and thus offers a more accurate representation of the scale’s reliability [32]. Following previous research, three models were tested: a unidimensional model, representing all items by a single factor; a two-factor model, that distinguishes between a subclinical factor (items 1–7) and a clinical or psychopathological factor (items 8–16) [22]; a four-factor model that assigns factors as follows: items [1, 2, 3] to the “intrusive thoughts” factor, items [5, 6, 7] to the “vivid daydreams” factor, items [11–15] to the “multisensory HLEs” factor, and items [4,8–10,16] to the “auditory and visual (AV) HLEs” factor [14,28,29,33].

To retain an item, a factor loading of at least 0.32 is required [48]. As shown in Table 2, all items met this criterion in both the two-factor and four-factor models. However, the four-factor model demonstrated the best fit according to the standard fit indices and information criteria (AIC, BIC) (Table 3).

**Table 2:** Descriptive statistics for the items of LSHS-E with factor loading of confirmatory factor analysis (n = 428).

LSHS-E Item	Mean (SD)	Factor loading 2-factor model		Factor loading: 4-factor model			
		F1	F2	F1	F2	F3	F4
**1.** Passing thought	1.8 (1.3)	0.56		0.72			
**2.** My thoughts seem as real as actual events	2.1 (1.4)	0.57		0.71			
**3.** Unrelated thoughts always creep into my mind	2.2 (1.4)	0.38		0.49			
**4.** Experience of hearing a person’s voice	1.6 (1.5)	0.60					0.60
**5.** Sounds I hear in my daydreams	1.4 (1.3)	0.81			0.81		
**6.** People in my daydreams seem so true to life	1.2 (1.4)	0.79			0.80		
**7.** My daydreams I can hear the sound of a tune	1.3 (1.4)	0.79			0.81		
**8.** Hear a voice speaking my thoughts aloud	1.2 (1.4)		0.73				0.76
**9.** Hearing voices in my head	1 (1.3)		0.74				0.74
**10.** Seen a person’s face in front of me	0.6 (1.1)		0.75				0.71
**11.** Experience of having seen, felt or heard something or someone that wasn’t there, or I had the feeling of being touched	0.7 (1.2)		0.74			0.76	
**12.** Felt that I was floating or falling, or that I was leaving my body temporarily	1.2 (1.5)		0.62			0.63	
**13.** Felt the presence of someone close who had passed away	0.6 (1.1)		0.65			0.64	
**14.** Smelt a particular odour	1.1 (1.4)		0.58			0.60	
**15.** Feeling of touching something or being touched	0.8 (1.3)		0.70			0.74	
**16.** Seen objects or animals	0.7 (1.2)		0.73				0.71

Two-factor (F1: subclinical and F2: clinical) and four-factor (F1: intrusive thoughts, F2: vivid daydreams, F3: multisensory HLEs and F4: auditory and visual HLEs) models are illustrated. The factor structure is based on previous literature [14,28,29,33].

**Table 3:** Goodness of fit indexes for the proposed models in the sample (n = 428).

Model	χ2	df	p	CFI	TLI	RMSEA (90%CI)	SRMR	AIC	BIC	McDonald’s omega
One-factor	491.66	104	0.001	0.86	0.84	0.09	0.07	20402.29	20532.19	0.92
Two-factor	378.96	103	0.001	0.90	0.89	0.08	0.06	20261.66	20395.61	0.93
Four-factor	302.61	98	0.001	0.93	0.91	0.07	0.05	20172.22	20326.46	0.93

CFI, comparative fit index; TLI, Tucker-Lewis Index; RMSEA, root mean square error of approximation; SRMR, standardized root mean square residual; AIC, Akaike information criterion; BIC, Bayesian information criterion.

### Convergent validation of LSHS-E

Convergent validity is defined by [49] as the correlation of independent methods measuring the same trait, with higher correlations indicating stronger validity. Using Spearman’s correlation, the results indicate significant positive correlations between HLEs measured by the LSHS-E, along with its subscales, and positive psychotic symptoms measured by the PCAPE. All correlations remained statistically significant after applying the Bonferroni correction. As shown in Table 4, the overall LSHS-E scores demonstrated a correlation coefficient of *r*_*s*_ = 0.55, *p < 0.001*. Among the subscales, intrusive thoughts (*r*_*s*_ *= 0.47, p < 0.001*), vivid daydreams (*r*_*s*_ = *0.42, p < 0.001*), multisensory hallucinatory experiences (*r*_*s*_ = 0.46, *p < 0.001*), and auditory-visual hallucinatory experiences (*r*_*s*_ = 0.52, *p < 0.001*) all showed high positive correlations with PCAPE. Notably, AV HLEs exhibited the strongest correlation (Table 4).

**Table 4:** Spearman correlation matrix between the LSHS-E, its factors, and PCAPE (n = 428).

	LSHS_E	Intrusive thoughts	Vivid daydreams	Multisensory HLEs	AV HLEs
**Intrusive thoughts**	0.73***				
**Vivid daydreams**	0.86***	0.54***			
**Multisensory HLEs**	0.83***	0.44***	0.59***		
**AV HLEs**	0.91***	0.53***	0.76***	0.73***	
**PCAPE**	0.55***	0.47***	0.42***	0.46***	0.52***

***p < 0.001.

### Effect of sociodemographic factors on the LSHS-E scores

Linear regression analyses revealed that age, independent financial status, income level, and social status were significant predictors of hallucination-proneness scores (Table 1). Younger participants scored higher on the LSHS-E compared to older participants (β = −5.30, *p*_*uncorrected*_ = 0.004, R^2^ = 0.03). Participants lacking independent financial income also reported higher scores (β = 3.50, *p*_*uncorrected*_ = 0.014, R^2^ = 0.01). Income showed a clear effect, with individuals in higher income brackets scoring significantly lower than those earning less (β = −6.92, *p*_*uncorrected*_ = 0.004, R^2^ = 0.04). Social status was also significant, with married participants scoring lower than single/separated/widowed participants (β = 3.24, *p*_*uncorrected*_ = 0.048, R^2^ = 0.01). In contrast, gender (β = 0.08, *p*_*uncorrected*_ = 0.957), education level (β = −3.85, *p*_*uncorrected*_ = 0.522), and employment status (β = 1.95, *p*_*uncorrected*_ = 0.152) were not significant predictors of hallucination proneness. Notably, after Bonferroni correction only age and income remained significant, while independent financial status and social status did not (Table 1). The variance explained by the models was modest, with the largest effects observed for income, followed by age.

## Discussion

This study aimed to validate the Arabic version of the LSHS-E and to explore the dimensional structure of HLEs in an Arabic-speaking, nonclinical population. By focusing on the general population and excluding factors such as psychopathology, alcohol, and drug use, we sought a clear assessment of HLEs within a normative setting. Based on previous studies, we hypothesized that the four-factor model observed in other languages would apply similarly in this cultural context. A large, diverse sample (n = 428) from the Saudi general population completed the LSHS-E, making the findings more representative than student-focused studies. Our analyses demonstrated that the Arabic LSHS-E showed strong reliability and convergent validity with the PCAPE, supported a four-factor structure consistent with prior research, and revealed that younger age and lower income were significant predictors of hallucination proneness. These findings align with evidence that a substantial proportion of the general population may experience HLEs or psychotic-like experiences at some point in their lives [7,41].

The Arabic LSHS-E demonstrated high internal consistency, as evidenced by Cronbach’s alpha, indicating its reliability as a measure of hallucination proneness in Arabic-speaking populations. Furthermore, the correlation between the LSHS-E, its four factors and the positive dimension of the CAPE showed strong convergent validity, suggesting that both tools effectively capture overlapping constructs related to hallucinations and positive psychotic symptoms.

CFA showed that the LSHS-E is best represented by four interrelated factors, replicating the multidimensional structure of hallucination proneness across Arabic-speaking populations. This finding aligns with the multidimensional models proposed in previous studies, suggesting that the construct is not only relevant but also reproducible across diverse languages and cultures. Though previous research has verified the multidimensional nature of the LSHS in different formats [9,16,18,22,27,30,31], factor structures often vary depending on the scale version used. Cross-cultural validations, such as the Persian version of the LSHS [30], further demonstrate the adaptability of the scale and reinforce the robustness of its multidimensional framework in non-Western populations.

While some research has supported a three-factor structure for the 16-item LSHS-E [27], our results align closely with the four-factor model proposed by previous studies, classifying HLEs into (1) intrusive thoughts, (2) vivid daydreams, (3) multisensory HLEs, and (4) AV HLEs [14,28,29,32,33]. These results support the four-factor conceptualization of HLEs as a stable structure within the general population.

Comparisons between the factor models in our results suggest that while both the two-factor and four-factor models showed acceptable fit and met the minimum factor loading criterion (Table 2), the latter demonstrated a slightly better fit across multiple indices. As shown in Table 3, the four-factor model yielded a higher CFI (0.93) compared to the two-factor model (0.90). Additionally, the four-factor model had a lower RMSEA (0.07) and SRMR (0.05), reflecting reduced residual error and a closer alignment with the observed data. Both the AIC (20172.22) and BIC (20326.46) were also lower for the four-factor model. These combined indices suggest that the four-factor model may better capture the dimensionality of HLEs in Arabic-speaking populations.

In terms of HLEs frequency, intrusive thoughts were among the most commonly reported experiences, consistent with findings from Indian [28], Italian [33], and Portuguese [27] studies, as well as the fundamental research by Bentall and Slade (1985b). For example, 41% of our sample reported that ‘Sometimes, a passing thought will seem so real that it frightens me’, comparable to the 59% observed in Sahu et al. (2020). Similarly, 52% of our participants endorsed the item ‘Sometimes, my thoughts seem as real as actual events in my life,’ compared to 57% in the Indian study, and 55% endorsed the item ‘No matter how hard I try to concentrate on my work, unrelated thoughts always creep into my mind,’ versus 59% in Sahu et al. (2020).

A similar pattern emerged between this study and Larøi and Van Der Linden’s (2005) original study, which identified five factors. Specifically, our findings for ‘intrusive thoughts’ and ‘vivid daydreams’ align closely with their structure, as these factors included the same items highlighted in Larøi and Van Der Linden’s (2005) work. This overlap may represent experiences that are distinguishable from other types of hallucinations. An alternative explanation might be that initial items are broadly endorsed across different populations, while endorsement rates tend to decline for items presented later in the scale.

Following intrusive thoughts, auditory hallucinations were also commonly reported, with 34% endorsing the item ‘In the past, I have had the experience of hearing a person’s voice and then found that there was no one there’. This result is comparable to those of previous studies, including Larøi and Van Der Linden (2005) (34%), Aleman et al. (2001) (31%), Waters et al. (2003) (28.5%), and Larøi et al. (2004) (28%). Conversely, visual hallucinations were less frequently reported, with items 10 (10.5%) and 16 (11.9%) endorsed at lower rates, a pattern consistent with Sahu et al. (2020) as well as the E-CLECTIC study [29] and the Portuguese study [27]. This contrasts with some studies indicated that visual hallucinations are commonly reported among healthy individuals [10,13], suggesting that the frequency of hallucinations across different sensory modalities may vary among nonclinical populations.

While the cross-sectional design of this study limits the ability to infer causality, the findings emphasise the role of demographic factors, such as age and socioeconomic status, as potential confounding variables in assessing hallucination proneness. In this sample, HLEs were negatively associated with age, with younger participants reporting higher scores on the LSHS-E. This aligns with previous studies showing that hallucination proneness is more prevalent among younger individuals [14,50–52]. Although the reasons behind this pattern are not fully understood, it has been suggested that older individuals may underreport such experiences due to concerns about stigma, being perceived as socially deviant, or misconceptions about hallucinations [29,53]. This hypothesis, while plausible, requires further empirical validation. Socioeconomic factors, particularly income also significantly influenced hallucination proneness, with individuals experiencing lower income levels reporting greater proneness. This association has been documented in prior research linking lower income levels to increased hallucination proneness [50] and a higher prevalence of psychotic symptoms [54]. In contrast, gender, education, marital status, independent financial status and professional status were not significant predictors in this study, differing from earlier research that has indicated these factors might moderate hallucination proneness [29]. This discrepancy potentially attributed to differences in sample characteristics, cultural factors, or methodological variations across studies.

There are several limitations to consider. Being a cross-sectional screening study, the primary aim was to determine if the LSHS-E would yield findings consistent with previous research, thus limiting the ability to draw strong conclusions about risk or mediational factors. Additionally, the study relied on online self-report measures rather than clinical assessments, which, despite the common use of this approach in research, introduces challenges in controlling for selection biases. However, self-report methods support the recruitment of large samples, and the assurance of data anonymity likely encouraged participants to respond more openly. Moreover, recruitment was conducted primarily through Saudi X accounts, and eligibility criteria specified that only Saudi individuals aged 18 years or older should participate. Although we were unable to independently confirm nationality or residence, which may represent a limitation of the study, other research has effectively employed online recruiting through social media targeting specific populations (e.g., Siddi et al., 2018). Finally, the exclusion of individuals who reported psychological or neurological disorders, or alcohol and drug use, means that the results cannot be generalised to clinical populations. Further validation in clinical samples is recommended to enhance the applicability of the findings.

## Implications

This study provides evidence for the multidimensional nature of hallucination proneness within an Arabic-speaking population, replicating findings from prior research. It valuably contributes to the existing body of work on psychotic symptoms in Arab populations specifically [41,55] and Eastern populations more broadly [30], where research has been comparatively limited relative to Western studies. Recent cross-cultural work has shown that although the prevalence of certain hallucinatory experiences may be lower in Middle Eastern samples (Qatari population) compared with European ones (Dutch population), their impact and clinical significance may be greater [55]. In this context, our data endorse the use of the Arabic translation of the LSHS-E questionnaire in future studies involving Arabic speakers. By developing and validating this version of the LSHS-E, we have created a reliable tool for continued research in this field.

## Conclusion

Taken together, this study provides a validated Arabic version of the LSHS-extended, enabling reliable screening for hallucination proneness among Arabic-speaking populations. The findings align with prior research across diverse cultural contexts, reinforcing the four-factor structure that characterizes the multidimensional nature of HLEs. Importantly, this work highlights that a notable proportion of the Saudi Arabian general population experiences HLEs, emphasising the prevalence of these phenomena beyond clinical settings.

## Supporting information

S1 Appendix Launay-Slade Hallucination Scale extended version (LSHS-E). English and Arabic versions of the questionnaire.
(DOCX)

S2 Appendix Positive subscale of the Community Assessment of Psychic Experiences (PCAPE) – 20 items. English and Arabic versions of the questionnaire.
(DOCX)

S3 Appendix Demographic questions. English and Arabic versions of the demographic questionnaire, with original and transformed sociodemographic categories.
(DOCX)

S4 Appendix Additional statistical analysis. Exploratory factor analysis (EFA).
(DOCX)

S5 Appendix Additional sociodemographic data analysis. (DOCX)

S1 Table Factor loadings for the two-factor model. (DOCX)

S2 Table Factor loadings for the four-factor model. (DOCX)

S3 Table Exploratory factor analysis results for two- and four-factor models and goodness of fit indexes. (DOCX)

S4 Table Multivariate regressions for Model 1 (age alone) and Model 2 (all variables) showing parameter estimates as well as std. error and p values (in brackets). (DOCX)

S5 Table Analysis of variance. (DOCX)

## References

[1] Slade PD, Bentall RP. Sensory deception: a scientific analysis of hallucination. Johns Hopkins University Press. 1988.

[2] Larøi F. How do auditory verbal hallucinations in patients differ from those in non-patients?. Front Hum Neurosci. 2012;6:25. doi: 10.3389/fnhum.2012.00025 22375112 PMC3282922

[3] Waters F, Allen P, Aleman A, Fernyhough C, Woodward TS, Badcock JC, et al. Auditory hallucinations in schizophrenia and nonschizophrenia populations: a review and integrated model of cognitive mechanisms. Schizophr Bull. 2012;38(4):683–93. doi: 10.1093/schbul/sbs045 22446568 PMC3406530

[4] Bentall RP. Madness explained: Psychosis and human nature. Penguin UK; 2004.

[5] American Psychiatric Association. Diagnostic and statistical manual of mental disorders: DSM-5: American psychiatric association; 2013.

[6] Bentall RP. The illusion of reality: a review and integration of psychological research on hallucinations. Psychol Bull. 1990;107(1):82–95. doi: 10.1037/0033-2909.107.1.82 2404293

[7] Bentall RP, Slade PD. Reliability of a scale measuring disposition towards hallucination: a brief report. Person Indi Diff. 1985;6(4):527–9. doi: 10.1016/0191-8869(85)90151-5

[8] Johns LC, van Os J. The continuity of psychotic experiences in the general population. Clin Psychol Rev. 2001;21(8):1125–41. doi: 10.1016/s0272-7358(01)00103-9 11702510

[9] Larøi F, Van Der Linden M. Nonclinical Participants’ Reports of Hallucinatory Experiences. Canadian J Behav Sci / Revue canadienne des sciences du comportement. 2005;37(1):33–43. doi: 10.1037/h0087243

[10] Sommer IEC, Daalman K, Rietkerk T, Diederen KM, Bakker S, Wijkstra J, et al. Healthy individuals with auditory verbal hallucinations; who are they? Psychiatric assessments of a selected sample of 103 subjects. Schizophr Bull. 2010;36(3):633–41. doi: 10.1093/schbul/sbn130 18849293 PMC2879692

[11] Temmingh H, Stein DJ, Seedat S, Williams DR. The prevalence and correlates of hallucinations in a general population sample: findings from the South African Stress and Health Study. Afr J Psychiatry (Johannesbg). 2011;14(3):211–7. doi: 10.4314/ajpsy.v14i3.4 21863206 PMC5638035

[12] Yates K, Lång U, Peters EM, Wigman JTW, McNicholas F, Cannon M, et al. Hallucinations in the general population across the adult lifespan: prevalence and psychopathologic significance. Br J Psychiatry. 2021;219(6):652–8. doi: 10.1192/bjp.2021.100 35048871

[13] Ohayon MM. Prevalence of hallucinations and their pathological associations in the general population. Psychiatry Res. 2000;97(2–3):153–64. doi: 10.1016/s0165-1781(00)00227-4 11166087

[14] Preti A, Sisti D, Rocchi MBL, Siddi S, Cella M, Masala C, et al. Prevalence and dimensionality of hallucination-like experiences in young adults. Compr Psychiatry. 2014;55(4):826–36. doi: 10.1016/j.comppsych.2014.01.015 24630201

[15] Launay G, Slade P. The measurement of hallucinatory predisposition in male and female prisoners. Person Ind Differ. 1981;2(3):221–34. doi: 10.1016/0191-8869(81)90027-1

[16] Larøi F, Marczewski P, Van der Linden M. Further evidence of the multi-dimensionality of hallucinatory predisposition: factor structure of a modified version of the Launay-Slade Hallucinations Scale in a normal sample. Eur Psychiatry. 2004;19(1):15–20. doi: 10.1016/S0924-9338(03)00028-2 14969776

[17] Morrison AP, Wells A, Nothard S. Cognitive factors in predisposition to auditory and visual hallucinations. Br J Clin Psychol. 2000;39(1):67–78. doi: 10.1348/014466500163112 10789029

[18] Aleman A, Nieuwenstein MR, Böcker KBE, De Haan EHF. Multi-dimensionality of hallucinatory predisposition: factor structure of the Launay–Slade Hallucination Scale in a normal sample. Person Ind Differ. 2001;30(2):287–92. doi: 10.1016/s0191-8869(00)00045-3

[19] Alganami F, Varese F, Wagstaff GF, Bentall RP. Suggestibility and signal detection performance in hallucination-prone students. Cogn Neuropsychiatry. 2017;22(2):159–74. doi: 10.1080/13546805.2017.1294056 28253093

[20] Bentall RP, Slade PD. Reality testing and auditory hallucinations: a signal detection analysis. Br J Clin Psychol. 1985;24(3):159–69. 4052663 10.1111/j.2044-8260.1985.tb01331.x

[21] El Haj M, Badcock JC, Larøi F. Hallucinations and source monitoring in Alzheimer’s disease. Cogn Neuropsychiatry. 2020;25(6):435–46. doi: 10.1080/13546805.2020.1832457 33043861

[22] Serper M, Dill CA, Chang N, Kot T, Elliot J. Factorial structure of the hallucinatory experience: continuity of experience in psychotic and normal individuals. J Nerv Ment Dis. 2005;193(4):265–72. doi: 10.1097/01.nmd.0000158374.54513.a0 15805823

[23] Levitan C, Ward PB, Catts SV, Hemsley DR. Predisposition toward auditory hallucinations: the utility of the Launay-Slade Hallucination Scale in psychiatric patients. Person Ind Differ. 1996;21(2):287–9. doi: 10.1016/0191-8869(96)00052-9

[24] Bellido-Zanin G, Perona-Garcelán S, Senín-Calderón C, López-Jiménez AM, Ruiz-Veguilla M, Rodríguez-Testal JF. Childhood memories of threatening experiences and submissiveness and its relationship to hallucination proneness and ideas of reference: The mediating role of dissociation. Scand J Psychol. 2018;59(4):407–13. doi: 10.1111/sjop.12455 29808908

[25] Diederen KMJ, De Weijer AD, Daalman K, Blom JD, Neggers SFW, Kahn RS, et al. Decreased language lateralization is characteristic of psychosis, not auditory hallucinations. Brain. 2010;133(Pt 12):3734–44. doi: 10.1093/brain/awq313 21097491

[26] Powers AR, van Dyck LI, Garrison JR, Corlett PR. Paracingulate Sulcus Length Is Shorter in Voice-Hearers Regardless of Need for Care. Schizophr Bull. 2020;46(6):1520–3. doi: 10.1093/schbul/sbaa067 32432706 PMC7707078

[27] Castiajo P, Pinheiro AP. On “Hearing” Voices and “Seeing” Things: Probing Hallucination Predisposition in a Portuguese Nonclinical Sample with the Launay-Slade Hallucination Scale-Revised. Front Psychol. 2017;8:1138. doi: 10.3389/fpsyg.2017.01138 28744234 PMC5504178

[28] Sahu S, Sharma V, Siddi S, Preti A, Malik D, Singhania S, et al. Validation of the Launay-Slade Hallucination Scale among Indian Healthy Adults. Asian J Psychiatr. 2020;53:102357. doi: 10.1016/j.ajp.2020.102357 32927310 PMC7935667

[29] Siddi S, Ochoa S, Laroi F, Cella M, Raballo A, Saldivia S, et al. A Cross-National Investigation of Hallucination-Like Experiences in 10 Countries: The E-CLECTIC Study. Schizophr Bull. 2019;45(Suppl 1):S43–55. doi: 10.1093/schbul/sby156 30715543 PMC6357978

[30] Goodarzi MA. Psychometric properties of a Persian translation of the Launay-Slade Hallucination Scale in an Iranian population. Percept Mot Skills. 2009;109(3):911–23. doi: 10.2466/pms.109.3.911-923 20178290

[31] Waters FAV, Badcock JC, Maybery MT. Revision of the factor structure of the Launay–Slade Hallucination Scale (LSHS-R). Personality and Individual Differences. 2003;35(6):1351–7. doi: 10.1016/s0191-8869(02)00354-9

[32] Siddi S, Ochoa S, Farreny A, Brébion G, Larøi F, Cuevas-Esteban J, et al. Measurement invariance of the Spanish Launay-Slade Hallucinations Scale-Extended version between putatively healthy controls and people diagnosed with a mental disorder. Int J Methods Psychiatr Res. 2018;27(4):e1741. doi: 10.1002/mpr.1741 30238666 PMC6877181

[33] Vellante M, Larøi F, Cella M, Raballo A, Petretto DR, Preti A. Hallucination-like experiences in the nonclinical population. J Nerv Ment Dis. 2012;200(4):310–5. doi: 10.1097/NMD.0b013e31824cb2ba 22456584

[34] Stefanis NC, Hanssen M, Smirnis NK, Avramopoulos DA, Evdokimidis IK, Stefanis CN, et al. Evidence that three dimensions of psychosis have a distribution in the general population. Psychol Med. 2002;32(2):347–58. doi: 10.1017/s0033291701005141 11866327

[35] Mark W, Toulopoulou T. Psychometric Properties of “Community Assessment of Psychic Experiences”: Review and Meta-analyses. Schizophr Bull. 2016;42(1):34–44. doi: 10.1093/schbul/sbv088 26150674 PMC4681550

[36] Therman S, Suvisaari J, Hultman CM. Dimensions of psychotic experiences among women in the general population. Int J Methods Psychiatr Res. 2014;23(1):62–8. doi: 10.1002/mpr.1427 24375586 PMC6878595

[37] IBM Corp. IBM SPSS Statistics for Windows, Version 27.0. Armonk, NY: IBM Corp. 2020.

[38] R Core Team. R: A language and environment for statistical computing. R Foundation for Statistical Computing; 2023.

[39] Rosseel Y. lavaan: AnRPackage for Structural Equation Modeling. J Stat Soft. 2012;48(2). doi: 10.18637/jss.v048.i02

[40] Jorgensen TD, Pornprasertmanit S, Schoemann AM, Rosseel Y, Miller P, Quick C, et al. semTools: Useful tools for structural equation modeling. R package version 05. 2018;1.

[41] Khaled SM, Wilkins SS, Woodruff P. Lifetime prevalence and potential determinants of psychotic experiences in the general population of Qatar. Psychol Med. 2020;50(7):1110–20. doi: 10.1017/S0033291719000977 31133090 PMC7253618

[42] DeVellis RF, Thorpe CT. Scale development: Theory and applications. Sage Publications. 2021.

[43] Kline RB. Principles and Practice of Structural Equation Modeling. Canad Stud Population. 2018;45(3–4):188–95.

[44] Curran PJ, West SG, Finch JF. The robustness of test statistics to nonnormality and specification error in confirmatory factor analysis. Psychol Method. 1996;1(1):16–29. doi: 10.1037/1082-989x.1.1.16

[45] Hu L, Bentler PM. Cutoff criteria for fit indexes in covariance structure analysis: Conventional criteria versus new alternatives. Struct Equation Model. 1999;6(1):1–55. doi: 10.1080/10705519909540118

[46] Akaike H. Factor Analysis and AIC. Psychometrika. 1987;52(3):317–32. doi: 10.1007/bf02294359

[47] Schwarz G. Estimating the Dimension of a Model. Ann Statist. 1978;6(2):461–4. doi: 10.1214/aos/1176344136

[48] Comrey A, Lee H. Interpretation and application of factor analytic results. Comrey AL, Lee HB A first course in factor analysis. 1992;2:1992.

[49] Dicken CF. Convergent and Discriminant Validity of the California Psychological Inventory. Edu Psychol Measure. 1963;23(3):449–59. doi: 10.1177/001316446302300303

[50] Pignon B, Schürhoff F, Szöke A, Geoffroy PA, Jardri R, Roelandt J-L, et al. Sociodemographic and clinical correlates of psychotic symptoms in the general population: Findings from the MHGP survey. Schizophr Res. 2018;193:336–42. doi: 10.1016/j.schres.2017.06.053 28689754

[51] Thompson R, Hallas L, Moseley P, Alderson-Day B. Cognitive and phenomenological characteristics of hallucination-proneness across the lifespan. Cogn Neuropsychiatry. 2021;26(1):18–34. doi: 10.1080/13546805.2020.1850435 33238807

[52] Larøi F, Bless JJ, Laloyaux J, Kråkvik B, Vedul-Kjelsås E, Kalhovde AM, et al. An epidemiological study on the prevalence of hallucinations in a general-population sample: Effects of age and sensory modality. Psychiatry Res. 2019;272:707–14. doi: 10.1016/j.psychres.2019.01.003 30832190

[53] Badcock JC, Dehon H, Larøi F. Hallucinations in Healthy Older Adults: An Overview of the Literature and Perspectives for Future Research. Front Psychol. 2017;8. 10.3389/fpsyg.2017.01134 PMC5500657 28736541

[54] Saha S, Scott JG, Varghese D, McGrath JJ. Socio-Economic Disadvantage and Delusional-Like Experiences: A Nationwide Population-Based Study. European Psychiatry. 2013;28(1):59–63. 22153729 10.1016/j.eurpsy.2011.09.004

[55] Khaled SM, Brederoo SG, Yehya A, Alabdulla M, Woodruff PW, Sommer IEC. Cross-cultural Differences in Hallucinations: A Comparison Between Middle Eastern and European Community-Based Samples. Schizophr Bull. 2023;49(12 Suppl 2):S13–24. doi: 10.1093/schbul/sbac086 36840542 PMC9960011

