# Supplementary material for: Validation of the Arabic version of the Launay-Slade Hallucination Scale Extended: A population-based online survey in Saudi-Arabia
Source: PLoS One. 2026 Feb 11;21(2):e0341864. doi: 10.1371/journal.pone.0341864 (PMC12893576; doi:10.1371/journal.pone.0341864)
Supplement: S1 Appendix — English and Arabic versions of the questionnaire. (DOCX) [file pone.0341864.s001.docx]

**Supporting information**

**S1 Appendix. Launay-Slade Hallucination Scale extended version (LSHS-E).** English and Arabic versions of the questionnaire.

The following statements describe some experiences that you may had in the past or face in your daily life, please indicate how much these sentences apply to you.

العبارات التالية تصف بعض الخبرات (المواقف) التي قد تكون تعرضت لها سابقاً أو تواجهها في حياتك اليومية، فضلا أجب بوضع دائرة على الرقم الذي يمثل درجة ملائمة العبارة لك (فضلاً اختر مدى تطابق هذه الاحداث معك)

**Answers**

- Certainly does not apply to me - مؤكد انها لا تنطبق علي
- Possibly does not apply to me - ربما لا تنطبق علي
- Unsure - غير متأكد
- Possibly applies to me - ربما تنطبق علي
- Certainly applies to me - مؤكد انها تنطبق علي

| **Items** |
| --- |
| 1. Sometimes a passing thought will seem so real that it frightens me   أحياناً فكرة عابرة تبدو لي حقيقية جداً لدرجة أنها تخيفني |
| 1. Sometimes my thoughts seem as real as actual events in my life   أحياناً أفكاري تبدو حقيقة كأنها أحداث واقعية في حياتي |
| 1. No matter how hard I try to concentrate on my work unrelated thoughts always creep into my mind   مهما حاولت جاهدا التركيز على عملي، دائماً تتسلل الى ذهني أفكار ليس لها علاقة |
| 1. In the past I have had the experience of hearing a person’s voice and then found that there was no-one there   في الماضي مررت بتجربة سماع صوت شخص ما، ثم اكتشفت أنه لم يكن هناك أحد |
| 1. The sounds I hear in my daydreams are generally clear and distinct   الأصوات التي أسمعها في أحلام اليقظة واضحة ومميزة بشكل عام |
| 1. The people in my daydreams seem so true to life that I sometimes think that they are   يبدو الأشخاص الذين أراهم في أحلام اليقظة وكأنهم حقيقيون لدرجة تجعلني أحيانا أعتقد كأنهم كذلك |
| 1. In my daydreams I can hear the sound of a tune almost as clearly as if I were actually listening to it   يمكنني سماع صوت نغمة بوضوح في أحلام اليقظة وكأنني أستمع اليه بالفعل |
| 1. I often hear a voice speaking my thoughts aloud   كثيرًا ما أسمع صوتًا عاليًا ينطق بأفكاري |
| 1. I have been troubled by hearing voices in my head   أُعاني من سماع أصوات في ذهني |
| 1. On occasions I have seen a person’s face in front of me when no-one was in fact there   أحيانًا أرى وجه شخص أمامي رغم عدم وجوده في الواقع |
| 1. Sometimes, immediately prior to falling asleep or upon awakening, I have had the experience of having seen, felt or heard something or someone that wasn’t there, or I had the feeling of being touched even though no one was there   أحيانًا، قبل النوم مباشرة أو عند الاستيقاظ، أرى أو أسمع أو أشعر بوجود شيئًا ما أو شخصًا ما غير موجود، أو أشعر بأن أحدًا يلمسني رغم عدم وجوده في الواقع |
| 1. Sometimes, immediately prior to falling asleep or upon awakening, I have felt that I was floating or falling, or that I was leaving my body temporarily   أحيانًا، قبل النوم مباشرة أو عند الاستيقاظ مباشرة، أشعر أنني أعوم أو أسقط، أو أغادر جسدي مؤقتًا |
| 1. On certain occasions I have felt the presence of someone close who had passed away   في مناسبات معينة، أشعر بتواجد شخص مقرب لي مع العلم أن هذا الشخص متوفى |
| 1. In the past, I have smelt a particular odour even though there was nothing there   في الماضي، شممت رائحة معينة رغم عدم وجودها في الواقع |
| 1. I have had the feeling of touching something or being touched and then found that nothing or no one was there   سبق وأن شعرت بأني ألمس شيئًا أو أن شيئًا يلمسني رغم عدم وجوده أو وجود أحد في الواقع |
| 1. Sometimes, I have seen objects or animals even though there was nothing there   أحيانا أرى أشياء أو حيوانات رغم عدم وجودها في الواقع |
