# Supplementary material for: Validation of the Arabic version of the Launay-Slade Hallucination Scale Extended: A population-based online survey in Saudi-Arabia
Source: PLoS One. 2026 Feb 11;21(2):e0341864. doi: 10.1371/journal.pone.0341864 (PMC12893576; doi:10.1371/journal.pone.0341864)
Supplement: S2 Appendix — English and Arabic versions of the questionnaire. (DOCX) [file pone.0341864.s002.docx]

**S2 Appendix. Positive subscale of the Community Assessment of Psychic Experiences (PCAPE) - 20 items.** English and Arabic versions of the questionnaire.

This questionnaire is created to measure specific emotions, perceptions and mental experiences. We believe these are much more common than previously thought, and that most people have had such experiences in their lifetime.

Please answer the following questions as truthfully as possible.

In the following questions, we ask you to highlight how often you have had special experiences during your life (the frequency of these experiences ranging from never to nearly always). You do this through choosing the answer that suits you best. There are no right or wrong answers.

تم إنشاء هذا الاستبيان لقياس مشاعر وحالات وتصورات وخبرات ذهنية محددة، من المُرجح أن هذه الخبرات أصبحت أكثر شيوعاً عنما كان يعتقد سابقًا، وأن معظم الناس قد مروا بمثل هذه التجارب في حياتهم.

الرجاء الإجابة على الأسئلة التالية بكل مصداقية ودقة ووضوح قدر الامكان.

في الأسئلة التالية، نطلب منك الإجابة على مدى تكرار هذه التجارب في حياتك، حيث أن مطلقاً (تعني أنك لم تمر بهذه المواقف ابدًا) ودائماً (تعني أنك دائماً تمر بهذه المواقف)، يمكنك القيام بذلك من خلال اختيار الإجابة التي تناسبك، مع العلم انه لا توجد اجابات صحيحة أو خاطئة.

**Answers**

- Never - مطلقًا
- Sometimes - أحيانًا
- Often - غالبًا
- Nearly always - دائما تقريبًا

| **Items** |
| --- |
| 2. Do you ever feel as if people seem to drop hints about you or say things with a double meaning?  هل شعرت يومًا وكأن الناس يسقطون تلميحات عنك أو يقولون كلمات ذات معاني مبطنة (تحتمل أكثر من معنى)؟  5. Do you ever feel as if things in magazines or on TV were written especially for you?  هل شعرت يومًا كما لو أن أشياء في الاعلام أو على شاشة التلفزيون تمت كتابتها خصيصًا لأجلك؟  6. Do you ever feel as if some people are not what they seem to be?  هل شعرت يومًا كما لو أن البعض لا يظهرون بصورتهم الحقيقية؟  7. Do you ever feel as if you are being persecuted in some way?  هل شعرت يومًا كما لو أنك تتعرض للاضطهاد بطريقة ما؟  10.  Do you ever feel as if there is a conspiracy against you?  هل شعرت يومًا وكأن هناك مؤامرة ضدك؟  11. Do you ever feel as if you are destined to be someone very important?  هل شعرت يومًا وكأنه مُقَدَّر لك أن تكون شخصًا مهمًا للغاية؟  13. Do you ever feel that you are a very special or unusual person?  هل شعرت يومًا أنك شخص مميز جدًا أو غير عادي؟  15. Do you ever think that people can communicate telepathically?  هل فكرت يومًا أن الناس يمكنهم التواصل عبر التخاطر الذهني؟  17. Do you ever feel as if electrical devices such as computers can influence the way you think?  هل شعرت يومًا أن الأجهزة الكهربائية مثل الكمبيوتر يمكن أن تؤثر على طريقة تفكيرك؟  20. Do you believe in the power of witchcraft, voodoo or the occult?  هل تؤمن بقوة السحر أوالشعوذة أو التنجيم؟  22. Do you ever feel that people look at you oddly because of your appearance?  هل شعرت يومًا أن الناس ينظرون إليك بغرابة بسبب مظهرك؟  24. Do you ever feel as if the thoughts in your head are being taken away from you?  هل شعرت يومًا وكأن الأفكار في رأسك يتم أخذها بعيداً عنك؟  26. Do you ever feel as if the thoughts in your head are not your own?  هل شعرت يومًا كما لو أن الأفكار في رأسك ليست ملكك (او ليست أفكارك)؟  28. Have your thoughts ever been so vivid that you were worried other people would hear them?  هل سبق وأن كانت أفكارك واضحة وظاهرة لدرجة أنك كنت قلقًا من أن يسمعها الآخرون؟  30. Do you ever hear your own thoughts being echoed back to you?  هل سبق لك أن سمعت صدى أفكارك وهي ترتد اليك؟  31. Do you ever feel as if you are under the control of some force or power other than yourself?  هل شعرت يومًا كما لو كنت تحت سيطرة قوة أخرى غيرك؟  33. Do you ever hear voices when you are alone?  هل سبق لك أن سمعت أصواتًا عندما تكون بمفردك؟  34. Do you ever hear voices talking to each other when you are alone?  هل سبق لك أن سمعت أصواتًا تتحدث مع بعضها البعض عندما تكون بمفردك؟  41. Do you ever feel as if a double has taken the place of a family member, friend or acquaintance?  هل شعرت يومًا كما لو أن شخصًا آخر قد حل محل أحد أفراد عائلتك أو أصدقائك أو أحد معارفك؟  42. Do you ever see objects, people or animals that other people cannot see?  هل سبق ورأيت أشياء أو أشخاصًا أو حيوانات لا يستطيع الآخرون رؤيتها؟ |
|  |

*Items are ordered according to the original full version of the CAPE.
