# Supplementary material for: Validation of the Arabic version of the Launay-Slade Hallucination Scale Extended: A population-based online survey in Saudi-Arabia
Source: PLoS One. 2026 Feb 11;21(2):e0341864. doi: 10.1371/journal.pone.0341864 (PMC12893576; doi:10.1371/journal.pone.0341864)
Supplement: S3 Appendix — English and Arabic versions of the demographic questionnaire, with original and transformed sociodemographic categories. (DOCX) [file pone.0341864.s003.docx]

***S3 Appendix. Demographic questions.*** *English and Arabic versions of the demographic questionnaire, with original and transformed sociodemographic categories.*

**Original sociodemographic coding**

| **Question (English)** | **السؤال (عربي)** |
| --- | --- |
| Gender   1. Female 2. Male | الجنس   1. أنثى 2. ذكر |
| Age group   1. 18–25 years 2. 26–35 years 3. 36–45 years 4. 46–55 years 5. 56–65 years 6. Above 65 years | الفئة العمرية   1. ١٨–٢٥ سنة 2. ٢٦–٣٥ سنة 3. ٣٦–٤٥ سنة 4. ٤٦–٥٥ سنة 5. ٥٦–٦٥ سنة 6. أكثر من ٦٥ سنة |
| Educational level   1. Illiterate 2. Elementary / Intermediate (up to middle school) 3. High school 4. University (Bachelor’s degree) 5. Postgraduate | المستوى التعليمي   1. أمي 2. ابتدائي / متوسط 3. ثانوي 4. جامعي (بكالوريوس) 5. دراسات عليا |
| Social status   1. Single 2. Married 3. Separated (divorced) 4. Widowed | الحالة الاجتماعية   1. أعزب / عزباء 2. متزوج / متزوجة 3. منفصل /منفصلة (مطلق) 4. أرمل / أرملة |
| Professional status (choose more than one if applicable)   1. Employed 2. Unemployed 3. Student 4. Free business (self-employed) 5. Retired | الحالة المهنية (يمكن اختيار أكثر من اجابة)   1. موظف / موظفة 2. عاطل / عاطلة 3. طالب / طالبة 4. عمل حر 5. متقاعد / متقاعدة |
| Do you have independent financial income?   1. Yes 2. No | هل لديك دخل مالي مستقل؟   1. نعم 2. لا |
| If yes, what is your average monthly income?   1. 4,000 riyals or less 2. 4,001 – 9,000 3. 9,001 – 14,000 4. 14,001 – 19,000 5. 19,001 – 24,000 6. 24,001 and above | إذا كانت الإجابة نعم، فما متوسط دخلك الشهري؟   1. ٤٠٠٠ ريال أو أقل 2. ٤٠٠١ – ٩٠٠٠ 3. ٩٠٠١ – ١٤٠٠٠ 4. ١٤٠٠١ – ١٩٠٠٠ 5. ١٩٠٠١ – ٢٤٠٠٠ 6. ٢٤٠٠١ فأكثر |
| Have you ever been diagnosed with a neurological disorder (e.g., Parkinson’s, Alzheimer’s, epilepsy)?   1. Yes 2. No | هل سبق أن تم تشخيصك باضطراب عصبي (مثل: باركنسون، الزهايمر، الصرع ... إلخ)؟   1. نعم 2. لا |
| Have you ever been diagnosed with a mental or psychological disorder (e.g., depression, schizophrenia, bipolar disorder, borderline personality disorder)?   1. Yes 2. No | هل سبق أن تم تشخيصك باضطراب نفسي أو عقلي (مثل: الاكتئاب، الفصام، الاضطراب ثنائي القطب، اضطراب الشخصية الحدية ... إلخ)؟   1. نعم 2. لا |
| Do you drink alcohol or use any type of drug?   1. Yes 2. No | هل تشرب الكحول أو تستخدم أي نوع من المخدرات؟   1. نعم 2. لا |

**Transformed categories**

| **Variable** |
| --- |
| Age group   1. 18–35 years 2. 36–55 years 3. 56 years or above |
| Educational level   1. Up to middle school 2. High school 3. Bachelor’s degree or higher |
| Social status   1. Married 2. Single, separated (divorced) or widowed |
| Professional status   1. Employed or free business (self-employed) 2. Unemployed, student or retired |
| Average monthly income   1. 9000 SR or less 2. 9001 – 19000 SR 3. Above 19000 SR |
