# Supplementary material for: Validation of the Arabic version of the Launay-Slade Hallucination Scale Extended: A population-based online survey in Saudi-Arabia
Source: PLoS One. 2026 Feb 11;21(2):e0341864. doi: 10.1371/journal.pone.0341864 (PMC12893576; doi:10.1371/journal.pone.0341864)
Supplement: S4 Appendix — Exploratory factor analysis (EFA). (DOCX) [file pone.0341864.s004.docx]

**S4 Appendix. Additional statistical analysis. Exploratory factor analysis (EFA).**

EFA of the Arabic LSHS-E with results for two- and four-factor models, including factor loadings (S1 and S2 Tables) and fit indices (S3 Table).

**Methods**

An EFA was conducted in R using the ‘psych’ package to examine the dimensional structure of the Arabic version of the LSHS-E. Both a 2-factor and a 4-factor model were tested using the minimum residual method (miners) with varimax rotation.

**Results**

As illustrated in S3 Table, the two-factor model explained 53% of the total variance, with factor 1 accounting for 29% and factor 2 for 25%. The model fit indices indicated reasonable fit (χ2(89) =415.73, p<1.1×10^-43^; RMSR = 0.04; RMSEA = 0.086, 90% CI (0.078, 0.095); TLI = 0.901; BIC = -135.76). The factor loadings (S1 Table) revealed that items largely clustered onto two factors, representing distinct dimensions of HLEs.

In comparison, the four-factor model (S2 and S3 Tables) provided a more detailed representation, explaining 59% of the total variance, with factor 1 contributing 22%, factor 2 contributing 13%, factor 3 contributing 8%, and factor 4 contributing 16%. This model demonstrated improved fit indices (χ2(62) =219.9, p<1.6×10^-19^ ; RMSR = 0.02; RMSEA = 0.072, 90% CI (0.062, 0.083); TLI = 0.931; BIC = -164.28).

Interestingly, the results of the two-factor model in the EFA are almost identical with the findings from the CFA except for item 8. In both analyses, the same items loaded strongly onto the two factors, and the variance explained was nearly identical. This may suggest the robustness of the two-factor structure and supports its generalizability within this dataset.
