# Supplementary material for: Validation of the Arabic version of the Launay-Slade Hallucination Scale Extended: A population-based online survey in Saudi-Arabia
Source: PLoS One. 2026 Feb 11;21(2):e0341864. doi: 10.1371/journal.pone.0341864 (PMC12893576; doi:10.1371/journal.pone.0341864)
Supplement: S5 Appendix — (DOCX) [file pone.0341864.s005.docx]

**S5 Appendix. Additional sociodemographic data analysis.**

Regression analyses and ANOVA models assessing sociodemographic predictors of LSHS-E scores.

The regression analysis for the individual sociodemographic factors showed that multiple variables, which are all substantially correlated, predict the LSHS-e score. Below are the parameter estimates for two separate multivariate analyses: Model 1 considers only the (highly significant) effect of age, while model 2 models all sociodemographic variables, including age (p=0.006) and finds average income to be (just) significant (p=0.045).
