# Supplementary material for: Validation of the Arabic version of the Launay-Slade Hallucination Scale Extended: A population-based online survey in Saudi-Arabia
Source: PLoS One. 2026 Feb 11;21(2):e0341864. doi: 10.1371/journal.pone.0341864 (PMC12893576; doi:10.1371/journal.pone.0341864)
Supplement: S1 Table — (DOCX) [file pone.0341864.s006.docx]

**S1 Table. Factor loadings for the two-factor model.**

| **Item** | **Factor 1** | **Factor 2** | **Communality (h^2^)** | **Uniqueness (u^2^)** | **Complexity** |
| --- | --- | --- | --- | --- | --- |
| 1 | 0.24 | **0.68** | 0.52 | 0.48 | 1.2 |
| 2 | 0.23 | **0.65** | 0.47 | 0.53 | 1.2 |
| 3 | 0.14 | **0.50** | 0.27 | 0.73 | 1.2 |
| 4 | 0.48 | **0.49** | 0.47 | 0.53 | 2.0 |
| 5 | 0.45 | **0.65** | 0.62 | 0.38 | 1.8 |
| 6 | 0.50 | **0.61** | 0.62 | 0.38 | 1.9 |
| 7 | 0.50 | **0.62** | 0.64 | 0.36 | 1.9 |
| 8 | 0.47 | **0.67** | 0.67 | 0.33 | 1.8 |
| 9 | **0.58** | 0.54 | 0.63 | 0.37 | 2.0 |
| 10 | **0.74** | 0.23 | 0.60 | 0.40 | 1.2 |
| 11 | **0.70** | 0.35 | 0.62 | 0.38 | 1.5 |
| 12 | **0.57** | 0.34 | 0.43 | 0.57 | 1.6 |
| 13 | **0.55** | 0.25 | 0.36 | 0.64 | 1.4 |
| 14 | **0.53** | 0.33 | 0.39 | 0.61 | 1.7 |
| 15 | **0.67** | 0.36 | 0.58 | 0.42 | 1.5 |
| 16 | **0.75** | 0.22 | 0.61 | 0.39 | 1.2 |
