# Supplementary material for: Validation of the Arabic version of the Launay-Slade Hallucination Scale Extended: A population-based online survey in Saudi-Arabia
Source: PLoS One. 2026 Feb 11;21(2):e0341864. doi: 10.1371/journal.pone.0341864 (PMC12893576; doi:10.1371/journal.pone.0341864)
Supplement: S2 Table — (DOCX) [file pone.0341864.s007.docx]

**S2 Table. Factor loadings for the four-factor model.**

| **Item** | **Factor 1** | **Factor 2** | **Factor 3** | **Factor 4** | **Communality (h^2^)** | **Uniqueness (u^2^)** | **Complexity** |
| --- | --- | --- | --- | --- | --- | --- | --- |
| 1 | 0.25 | **0.73** | 0.10 | 0.23 | 0.66 | 0.34 | 1.5 |
| 2 | 0.16 | **0.56** | 0.19 | 0.34 | 0.49 | 0.51 | 2.2 |
| 3 | 0.17 | **0.53** | 0.04 | 0.15 | 0.33 | 0.67 | 1.4 |
| 4 | **0.53** | 0.37 | 0.02 | 0.32 | 0.52 | 0.48 | 2.5 |
| 5 | 0.34 | 0.34 | 0.16 | **0.65** | 0.68 | 0.32 | 2.2 |
| 6 | 0.36 | 0.31 | 0.24 | **0.63** | 0.69 | 0.31 | 2.5 |
| 7 | 0.40 | 0.31 | 0.15 | **0.65** | 0.71 | 0.29 | 2.3 |
| 8 | 0.35 | 0.39 | 0.25 | **0.60** | 0.70 | 0.30 | 2.8 |
| 9 | **0.51** | 0.36 | 0.22 | 0.43 | 0.62 | 0.38 | 3.2 |
| 10 | **0.59** | 0.13 | 0.42 | 0.25 | 0.60 | 0.40 | 2.3 |
| 11 | **0.76** | 0.23 | 0.09 | 0.27 | 0.71 | 0.29 | 1.5 |
| 12 | **0.54** | 0.26 | 0.19 | 0.22 | 0.45 | 0.55 | 2.1 |
| 13 | 0.29 | 0.13 | **0.76** | 0.21 | 0.73 | 0.27 | 1.5 |
| 14 | **0.48** | 0.25 | 0.24 | 0.22 | 0.40 | 0.60 | 2.6 |
| 15 | **0.66** | 0.23 | 0.16 | 0.29 | 0.60 | 0.40 | 1.8 |
| 16 | **0.65** | 0.13 | 0.31 | 0.23 | 0.59 | 0.41 | 1.8 |
