# Supplementary material for: Validation of the Arabic version of the Launay-Slade Hallucination Scale Extended: A population-based online survey in Saudi-Arabia
Source: PLoS One. 2026 Feb 11;21(2):e0341864. doi: 10.1371/journal.pone.0341864 (PMC12893576; doi:10.1371/journal.pone.0341864)
Supplement: S3 Table — (DOCX) [file pone.0341864.s008.docx]

**S3 Table. Exploratory factor analysis results for two- and four-factor models and goodness of fit indexes.**

| **Model** | **Factor SS Loadings** | **Explained variance (%)** | **Cumulative variance (%)** | **RMSR** | **RMSEA (90% CI)** | **TLI** | **BIC** | **χ2** | **df** | **p** |
| --- | --- | --- | --- | --- | --- | --- | --- | --- | --- | --- |
| **Two-Factor** | F1: 4.57 F2: 3.93 | F1: 29% F2: 25% | 53% | 0.04 | 0.086 (0.078, 0.095) | 0.901 | -135.76 | 415.73 | 89 | <1.1×10^-43^ |
| **Four-Factor** | F1: 3.58 F2: 2.13 F3: 1.25 F4: 2.51 | F1: 22% F2: 13%  F3: 8% F4: 16% | 59% | 0.02 | 0.072 (0.062, 0.083) | 0.931 | -164.28 | 219.9 | 62 | <1.6×10^-19^ |

*RMSR, Root mean square of residuals; RMSEA, root mean square error of approximation; TLI, Tucker-Lewis index; and BIC; Bayesian information criterion.*
