# Supplementary material for: Validation of the Arabic version of the Launay-Slade Hallucination Scale Extended: A population-based online survey in Saudi-Arabia
Source: PLoS One. 2026 Feb 11;21(2):e0341864. doi: 10.1371/journal.pone.0341864 (PMC12893576; doi:10.1371/journal.pone.0341864)
Supplement: S4 Table — (DOCX) [file pone.0341864.s009.docx]

**S4 Table. Multivariate regressions for Model 1 (age alone) and Model 2 (all variables) showing parameter estimates as well as std. error and p values (in brackets).**

|  | **Model 1 (age only)** | **Model 2 (all vars)** |
| --- | --- | --- |
| (Intercept) | 25.386 | 33.918 |
|  | 1.739 (<0.001) | 7.057 (<0.001) |
| age group | **-1.987** | **-2.090** |
|  | **0.555 (<0.001)** | **0.752 (0.006)** |
| Gender |  | 3.450 |
|  |  | 1.768 (0.052) |
| education level |  | -1.927 |
|  |  | 1.087 (0.077) |
| social status |  | -0.106 |
|  |  | 1.550 (0.946) |
| professional status |  | 0.461 |
|  |  | 0.562 (0.413) |
| financial status |  | -2.168 |
|  |  | 2.290 (0.344) |
| average income |  | **-1.355** |
|  |  | **0.674 (0.045)** |
| Num.Obs. | 428 | 428 |
| R2 | 0.029 | 0.070 |
| R2 Adj. | 0.027 | 0.055 |
| AIC | 3470.3 | 3463.8 |
| BIC | 3482.5 | 3500.4 |
| Log.Lik. | -1732.150 | -1722.917 |
| RMSE | 13.85 | 13.55 |
