# Supplementary material for: Validation of the Arabic version of the Launay-Slade Hallucination Scale Extended: A population-based online survey in Saudi-Arabia
Source: PLoS One. 2026 Feb 11;21(2):e0341864. doi: 10.1371/journal.pone.0341864 (PMC12893576; doi:10.1371/journal.pone.0341864)
Supplement: S5 Table — (DOCX) [file pone.0341864.s010.docx]

**S5 Table. Analysis of variance.**

| **Model** | **Res.Df** | **RSS** | **Df** | **Sum of Sq** | **F** | **Pr(>F)** |
| --- | --- | --- | --- | --- | --- | --- |
| 1 | 426 | 82076 |  |  |  |  |
| 2 | 420 | 78611 | 6 | 3466 | 3.0863 | 0.0057 |

**Model 1:** LSHS-E score ~ age group

**Model 2:** LSHS-E score ~ Gender + age group + education level + social status + professional status + financial status + average income

The analysis of variance shows that the two model predictions differ significantly, suggesting that age, while being a dominant confound, is not the only factor.
